# Prevalence of Germline Pathogenic Variants in Cancer Predisposing Genes in Czech and Belgian Pancreatic Cancer Patients

**DOI:** 10.3390/cancers13174430

**Published:** 2021-09-02

**Authors:** Greet Wieme, Jan Kral, Toon Rosseel, Petra Zemankova, Bram Parton, Michal Vocka, Mattias Van Heetvelde, Petra Kleiblova, Bettina Blaumeiser, Jana Soukupova, Jenneke van den Ende, Petr Nehasil, Sabine Tejpar, Marianna Borecka, Encarna B. Gómez García, Marinus J. Blok, Marketa Safarikova, Marta Kalousova, Karen Geboes, Robin De Putter, Bruce Poppe, Kim De Leeneer, Zdenek Kleibl, Marketa Janatova, Kathleen B. M. Claes

**Affiliations:** 1 Center for Medical Genetics, Ghent University and Ghent University Hospital, 9000 Ghent, Belgium; Greet.Wieme@UGent.be (G.W.); Toon.Rosseel@UGent.be (T.R.); Bram.Parton@UGent.be (B.P.); Mattias.VanHeetvelde@UGent.be (M.V.H.); Robin.dePutter@UGent.be (R.D.P.); Bruce.Poppe@UGent.be (B.P.); Kim.DeLeeneer@UGent.be (K.D.L.); 2 Cancer Research Institute Ghent (CRIG), 9000 Ghent, Belgium; karen.geboes@uzgent.be; 3 Institute of Biochemistry and Experimental Oncology, First Faculty of Medicine, Charles University, 128 53 Prague, Czech Republic; jakral@lf1.cuni.cz (J.K.); pboud@lf1.cuni.cz (P.Z.); jana.soukupova@lf1.cuni.cz (J.S.); Petr.Nehasil@vfn.cz (P.N.); Marianna.Borecka@vfn.cz (M.B.); zdekleje@lf1.cuni.cz (Z.K.); 4 Department of Oncology, First Faculty of Medicine, Charles University and General University Hospital in Prague, 128 00 Prague, Czech Republic; Michal.Vocka@vfn.cz; 5 Institute of Biology and Medical Genetics, First Faculty of Medicine, Charles University and General University Hospital in Prague, 128 00 Prague, Czech Republic; pekleje@lf1.cuni.cz; 6 Center of Medical Genetics UZA/UA, Antwerp University Hospital, 2000 Antwerp, Belgium; bettina.blaumeiser@uantwerpen.be (B.B.); jenneke.vandenende@uantwerpen.be (J.v.d.E.); 7 Department of Paediatrics and Inherited Metabolic Disorders, First Faculty of Medicine, Charles University and General University Hospital in Prague, 128 00 Prague, Czech Republic; 8 Digestive Oncology Unit, University Hospital Gasthuisberg, 3000 Leuven, Belgium; sabine.tejpar@uzleuven.be; 9 Department of Clinical Genetics, Maastricht University Medical Centre, 6229 GR Maastricht, The Netherlands; encarna.gomezgarcia@mumc.nl (E.B.G.G.); rien.blok@mumc.nl (M.J.B.); 10 GROW School for Oncology & Developmental Biology, Maastricht University Medical Centre, 6200 MD Maastricht, The Netherlands; 11 Institute of Medical Biochemistry and Laboratory Diagnostics, First Faculty of Medicine, Charles University and General University Hospital in Prague, 128 00 Prague, Czech Republic; Marketa.Safarikova@vfn.cz (M.S.); marta.kalousova@lf1.cuni.cz (M.K.); 12 Department of Gastroenterology, Ghent University Hospital, 9000 Ghent, Belgium

**Keywords:** pancreatic ductal adenocarcinoma, overall survival, multigene panel testing, family history, germline

## Abstract

**Simple Summary:**

We performed genetic analysis of 53 cancer predisposing genes in Belgian and Czech pancreatic cancer patients. In known pancreatic cancer predisposing genes, a high mutation detection ratio was observed in patients with multiple primary tumors and/or a family history of pancreatic or breast, ovarian or colon cancer or melanoma. *BRCA1, BRCA2,* and *ATM* were most frequently affected. Pathogenic variants in cancer predisposition genes for which the association with pancreatic cancer has not been firmly established, were less frequent, except for *CHEK2*. This observation warrants further analyses in other populations. To accurately determine risk associations our study highlights the importance of using a geographically-matched control population.

**Abstract:**

(1) Background: The proportion and spectrum of germline pathogenic variants (PV) associated with an increased risk for pancreatic ductal adenocarcinoma (PDAC) varies among populations. (2) Methods: We analyzed 72 Belgian and 226 Czech PDAC patients by multigene panel testing. The prevalence of pathogenic variants (PV) in relation to personal/family cancer history were evaluated. PDAC risks were calculated using both gnomAD-NFE and population-matched controls. (3) Results: In 35/298 (11.7%) patients a PV in an established PDAC-predisposition gene was found. *BRCA1/2* PV conferred a high risk in both populations, *ATM* and Lynch genes only in the Belgian subgroup. PV in other known PDAC-predisposition genes were rarer. Interestingly, a high frequency of *CHEK2* PV was observed in both patient populations. PV in PDAC-predisposition genes were more frequent in patients with (i) multiple primary cancers (12/38; 32%), (ii) relatives with PDAC (15/56; 27%), (iii) relatives with breast/ovarian/colorectal cancer or melanoma (15/86; 17%) but more rare in sporadic PDAC (5/149; 3.4%). PV in homologous recombination genes were associated with improved overall survival (HR = 0.51; 95% CI 0.34–0.77). (4) Conclusions: Our analysis emphasizes the value of multigene panel testing in PDAC patients, especially in individuals with a positive family cancer history, and underlines the importance of population-matched controls for risk assessment.

## 1. Introduction

Pancreatic ductal adenocarcinoma (PDAC) remains a deadly malignancy with a 5-year survival rate of only 9% [1]. Its initial non-specific symptoms makes early diagnosis challenging and less than 20% of PDAC patients have potentially resectable tumors at the time of diagnosis [2]. Although most PDAC cases appear to be sporadic, a familial background has been documented in up to 10% of the patients [2,3]. In addition, increased PDAC prevalence associates with hereditary cancer syndromes caused by germline pathogenic/likely-pathogenic variants (PV) in *BRCA2, ATM, BRCA1, PALB2, MLH1, MSH2, MSH6, PMS2, CDKN2A, TP53*, or *STK11* [3,4]. Germline PV in the four first genes have the highest prevalence in PDAC patients; however, their frequency shows substantial geographic variabilities [5].

Individuals with PV in PDAC-predisposing genes could benefit from intensified preventive surveillance allowing early PDAC detection, when curative surgery may still be feasible [6]. Interestingly, germline PV in cancer susceptibility genes have also been reported in apparently sporadic PDAC cases [7,8]. Several recent reports noticed an improved PDAC prognosis in patients with germline PV in genes encoding proteins involved in DNA damage response (DDR) and, notably, those involved in homologous recombination (HR) to repair DNA double-strand breaks (DDSB) [9,10,11]. In addition, the presence of germline PV in these genes enables tailored treatment by poly(ADP-ribose)polymerase inhibitors (PARPi) [12,13]. The identification of PV allows for predictive testing in affected families to identify relatives at risk.

Multigene panel testing is the preferred method for the identification of germline PV in genetically heterogeneous disorders since it enables fast, reliable, and cost-effective analysis of single nucleotide variants (SNV), short insertions/deletions (indels), and may allow detection of copy number variants (CNV) in predisposition genes, after strong validation [14].

The purpose of this study was to determine and compare the prevalence of germline PV in cancer predisposing genes in identically evaluated sets of Belgian high-risk PDAC patients and Czech unselected PDAC patients. We also aimed to determine the subgroup of PDAC patients who may benefit from multigene panel testing.

## 2. Materials and Methods

### 2.1. Patients and Samples

Overall, 226 consecutive Czech PDAC patients (Table 1) diagnosed at the Dept. of Oncology, General University Hospital in Prague between 2015 and 2018 were enrolled. All patients gave informed consent for their participation, approved by the Ethics Committee of the General University Hospital in Prague. All patients were Caucasians of Czech origin. The Czech population-matched (PM) controls were described previously and represent 777 non-cancer volunteers aged >60 years that were analyzed identically as the Czech PDAC patients [15].

Additionally, 72 Belgian PDAC patients (Table 1) were retrospectively selected from the patient database of the Center for Medical Genetics at Ghent University Hospital (CMGG; *n* = 62) and other genetic centers in Belgium and the Netherlands (*n* = 10). Eligible individuals included PDAC patients fulfilling testing criteria for analysis of hereditary cancer syndromes. Patients were counseled between 2000 and 2019 by clinical geneticists of the center and had signed an informed consent agreeing to store their DNA and perform extra analyses in the context of their disease. This study was approved by the ethical committee of Ghent University Hospital. All patients have been selected from strata based on personal or family history (first- and second-degree relatives) of PDAC in combination with breast/ovarian/colon cancer and/or melanoma. Belgian PM controls represent anonymized whole exome sequencing data from 2485 unselected individuals sequenced in CMGG for various non-cancer conditions (intellectual disability, blindness, muscular dystrophies, cardiomyopathies).

### 2.2. NGS and Bioinformatics

Both centers applied their proprietary NGS panel overlapping in 53 target genes (Table S1). Pathogenic variants in these genes were identified subsequently using the unified prioritization described below.

Czech: Patients’ samples were analyzed as described previously [16]. Briefly, a custom-designed panel CZECANCA (CZEch CAncer paNel for Clinical Application; ver_1.2) was used to capture 226 genes (Table S2). DNA libraries were prepared using KAPA HTP Library Preparation kit (Roche, Basel, Switzerland) and sequenced on MiSeq/NextSeq500 (Illumina, San Diego, CA 92121, USA). NGS data were processed by NovoAlign and annotated by ANNOVAR. Copy number variations (CNVs) were analyzed by CNVkit, medium-size indels by Pindel.

Belgium: Genomic DNA samples were fragmented with the KAPA HyperPlus Library Preparation Kit (Roche). The regions of interest were captured by designed SeqCAP EZ probes targeting 66 genes (Table S3) and sequenced on MiSeq/NovaSeq6000 (Illumina, San Diego, CA 92121, USA). NGS data were processed using an in-house pipeline. The NGS reads were processed by the bcbio toolkit including data mapping to the reference genome by BWA, variant calling using VarDict, and variant annotation in Ensembl Variant Effect Predictor and dbNSFP/dbscSNV databases. Final quality control was performed by FastQC, samtools and bcftools. Coverage was analyzed using mosdepth.

### 2.3. Variant Prioritization

The same variant prioritization was applied at both sites to identify PV in coding and flanking intronic variants (±20 bp) in 53 targeted genes. The prioritization pipeline excluded variants:

- With low variant allele fraction (VAF < 0.15);
- With a high minor allele frequency (MAF > 0.001) in population databases: Exome Sequencing Project (ESP), 1000 Genomes Project and gnomAD, except variants classified pathogenic/likely pathogenic (P/LP) in ClinVar;
- In UTR, non-splice site intronic, synonymous and non-frameshift insertions/deletions, unless classified as P/LP in ClinVar;
- Classified as benign/likely benign (B/LB) in ClinVar, if marked by at least two stars in ClinVar or if classified as B/LB by an expert panel;
- Low risk variants in *CHEK2* (c.470C > T; p.I157T), *APC* (c.3920T > A; p.I1307K), and heterozygous *MUTYH* variants.

The remaining variants were classified in accordance with the ACMG recommendations [17]. Variants with insufficient or conflicting evidence were categorized as variants of uncertain significance (VUS) as well as truncating variants in the last exon (except ClinVar P/LP). All PV in patients were inspected in Integrative Genomics Viewer (IGV) or confirmed by Sanger sequencing and submitted to ClinVar and LOVD.

### 2.4. Statistical Analysis

The frequencies of PV in PDAC patients were compared to the frequencies of PV in both region-matched controls and the gnomAD control dataset with the non-Finnish European (NFE) exome data, from release 2.1.1 (restricted to gnomAD exome data) [18]. Associations of PDAC with germline PV in individual genes were analyzed using the Fisher’s exact test in different PDAC subgroups. The odds ratios (ORs) and corresponding 95% confidence intervals (CI) were calculated by inverting Fisher’s exact test. Bonferroni correction was applied to adjust p-values for the number of mutated genes within each population. All statistical tests were two-sided, and adjusted *p*-values < 0.05 were considered statistically significant. Association analyses were performed with R (version 3.6.1; The R Foundation).

The Kaplan-Meier product-limit method was used for survival analyses and differences were tested using the log-rank and Mantel-Haenszel tests using the GraphPad Prism v8.0.1 (GraphPad Software) and Statistica v12 (StatSoft) programs. The overall survival (OS) was defined as the interval between PDAC diagnosis and death from PDAC or the last follow-up.

## 3. Results

### 3.1. Spectrum and Frequencies of Germline Alterations in PDAC Patients

In total, we detected 61 germline PV in 20 out of 53 genes analyzed in both population cohorts, comprising 72 high-risk Belgian and 226 unselected Czech PDAC patients (Table 2 and Table S4). Thirty-six PV were found in “established PDAC-predisposition” genes (*BRCA2, ATM, BRCA1, PALB2, MLH1, MSH2, MSH6, PMS2, CDKN2A, TP53*, or *STK11* [3,4]) in 35/298 (11.7%) patients. Of these, 30 patients were found to have a PV in one PDAC-predisposition gene, one patient harbored a PV in both *MLH1* and *MSH6*, while four patients inherited a PV in a PDAC-predisposing and another gene (Table 2). *BRCA2* (4.0%), *ATM* (2.7%), and *BRCA1* (1.7%) were the most frequently mutated PDAC-predisposition genes. Additionally, in 23 PDAC patients (7.7%) 25 PV were found in other cancer predisposition genes for which a clear association with PDAC has not (yet) been established; one third of them in *CHEK2*.

**Figure 1:**
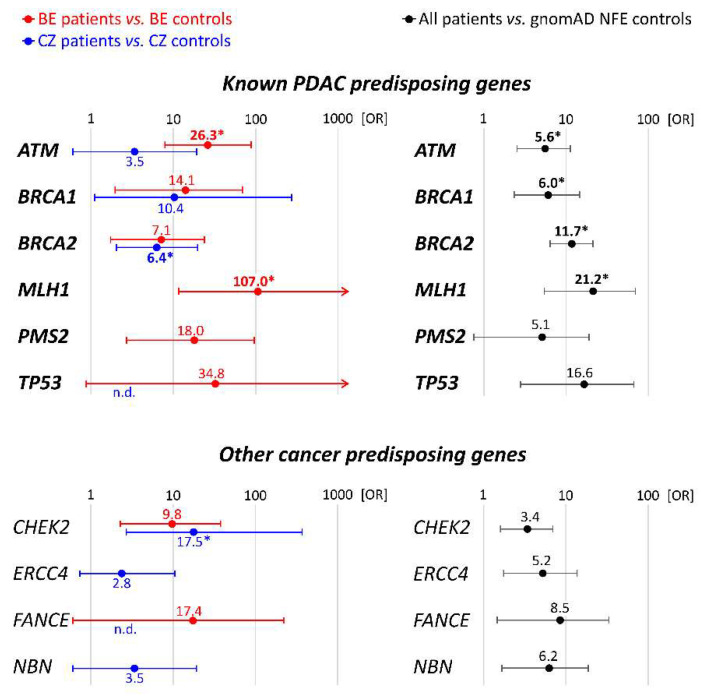
Gene specific PDAC risks in Belgian and Czech PDAC patients (compared with population-matched controls) and all PDAC patients (compared with gnomAD NFE). The plots showing OR and 95%CI describe only genes associated significantly with PDAC Belgian, Czech, or all PDAC patients, respectively. All genes are presented in detail in Table S5. OR values in bold denoted by an asterisk (*) remained significant following Bonferroni correction for multiple testing. n.d.—not determined (zero carriers in population-matched controls).

Gene-specific PDAC risks were calculated for Belgian and Czech patients separately using the respective population-matched controls and for the entire group using gnomAD NFE controls. Genes associated with a significant risk are displayed in Figure 1; risks for all genes with PV are summarized in Table S5.

Considering the established PDAC-predisposition genes, the population-specific PDAC risks were significantly increased for Belgian patients with PV in *ATM, BRCA1, BRCA2, MLH1,* and *PMS2* and for Czech patients with a PV in *BRCA1* and *BRCA2*. A comparison of all PDAC patients with gnomAD-NFE controls confirmed a PDAC risk association for all mentioned genes, except PMS2, but additionally revealed an association with *TP53*. For the other cancer predisposition genes, a significant association was found for *CHEK2* (with both population-matched and gnomAD controls) and for *ERCC4, FANCE*, and *NBN* (gnomAD controls only).

In view of the overrepresentation of high-risk PDAC patients in the Belgian group (significant excess of individuals with positive individual/familial cancer history; Table 1), the higher overall frequency of PV in PDAC-predisposition genes in Belgian over Czech PDAC patients (23.6% vs. 8.0%, respectively; *p* = 0.001) was not surprising. In contrast, the proportion of germline PV in other genes did not differ significantly between the Belgian and Czech patients (9.7% and 7.1%, respectively; *p* = 0.45).

### 3.2. Personal Cancer History

**Figure 2:**
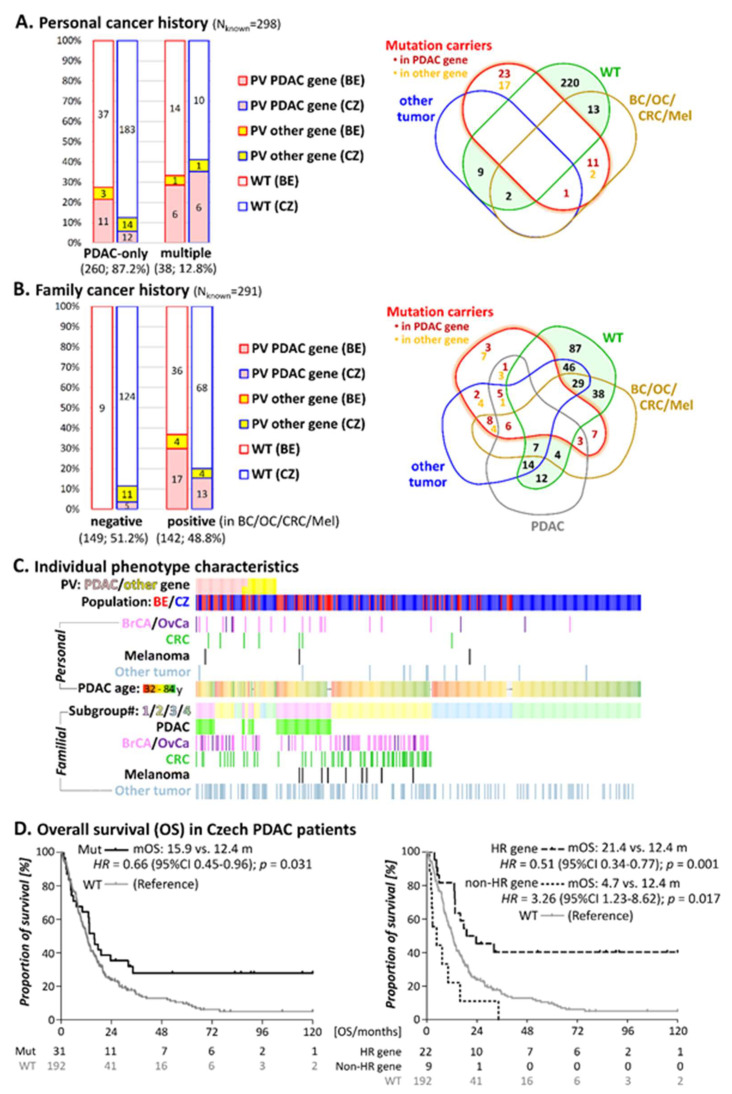
Proportions (left) of patients with PV classified according to personal (**A**) and family (**B**) cancer history considering the presence of PDAC, breast cancer (BC), ovarian cancer (OC), colorectal cancer (CRC) or melanoma in first or second degree relative. The types (right) of multiple primary tumors (**A**) and tumors in the first/second degree relatives (**B**) are illustrated in a Venn diagram. Individual phenotype characteristics (**C**) arranged subsequently according to the presence of PV, family cancer history positivity, presence of multiple primary tumors and the age of PDAC onset. Overall survival in the Czech PDAC patients (**D**) ascertained according to the presence of any germline PV (left) and in PV in HR and non-HR genes, respectively (right). The numbers below the graphs denote the numbers of individual in displayed time points.

PV in PDAC-predisposition genes were 4-times more frequent in patients with multiple primary tumors (12/38; 31.6%) than in PDAC-only patients (23/260; 8.8%; *p* < 0.001; Figure 2A). PV in 10 PDAC patients with multiple primary tumors affected 3 × *BRCA1* and 4 × *BRCA2* (all had also developed breast and/or ovarian cancer), 1 × *PMS2* and 1 × *MLH1/MSH6* (both developed colon cancer), and 1 × *CDKN2A* (diagnosed with melanoma). In addition, two patients harbored a PV in a PDAC-predisposition and another gene: *ATM/FANCE* (PDAC patient with colon cancer) and *BRCA2/CHEK2* (early-onset breast cancer PDAC patient). Furthermore, a pathogenic *NBN* variant was detected in a PDAC patient with breast cancer and a *POLE* PV in a PDAC patient who had also developed breast and colon cancer.

The proportion of PV carriers in PDAC-predisposition genes did not differ significantly between Belgian and Czech patients with multiple primaries (6/21, 28.6% and 6/17, 35.3%, respectively; Figure 2A), although the fraction of PDAC patients with multiple primary tumors was considerably higher in the Belgian over the Czech group (29.2% vs. 7.5%; Table 1). About one third (31.6%) of the PV in PDAC-predisposition genes (13/36) was identified in patients with multiple primaries, even when they accounted for only 12.8% of the total study population (Figure 2A). Thus, the presence of another primary tumor alongside the PDAC diagnosis increased the chance for a PV in a PDAC-predisposition gene. It is of note that all patients with double primary tumors and a PV in PDAC-predisposition genes, also had a positive family cancer history.

No significant difference in the age of PDAC onset could be established between patients with and without PV in PDAC-predisposition genes (with PV: mean 59.9 years; range 32–82 years; without PV: mean 62.2 years; range 37–84 years; *p* = 0.20). The same observation was done for patients with PV in other genes (mean 62.5 years; range 35–80 years) versus patients without PV (*p* = 0.92).

### 3.3. Family Cancer History

To overcome differences in clinical characteristics between Belgian and Czech PDAC patients, we assigned all patients into four subgroups (Table 3). Subgroup #1 included patients with a positive PDAC family cancer history. Subgroup #2 included patients with a family history of tumors (breast/ovary/colorectal cancer/melanoma) indicative for PDAC-associated hereditary cancer syndromes. Patients with a negative family cancer history or with non-syndromic tumors in relatives were assigned into additional subgroups according to the age of their PDAC onset (subgroup #3: ≤60 years; subgroup#4: >60 years). The family cancer history was not detailed enough for 6 Belgian and 1 Czech PDAC patients, hence the family cancer history was only taken into account for 291 PDAC-patients with a detailed familial anamneses.

In subgroups #1 and #2 (patients with a positive family cancer history) PV in PDAC-predisposition are much more prevalent than in subgroups #3 and #4 (sporadic) (subgroups #1 and #2: 30/142, 21.1% vs. subgroups #3 and #4: 5/149, 3.4%; *p* < 0.001) (Figure 2B and Table 3).

The proportion of patients heterozygous for PV in established PDAC-susceptibility genes in subgroup #1 (15/56; 26.8%) was similar in Belgian (26.5%) and Czech (27.3%) patients (Table 3) and concerned 5 × *BRCA2*, 3 × *ATM*, 3 × Lynch syndrome genes, 2 × *BRCA1*, 1 × *TP53* and 1 × *CDKN2A*. Additionally, three patients harbored the c.1100delC variant in *CHEK2* and another one had a PV in *FANCM*.

The prevalence of PV in established PDAC-susceptibility genes in subgroup #2 (15/86; 17.4%), was higher in Belgian (34.8%) than in Czech (11.1%) patients (*p* = 0.04; Figure 2B). However, this subgroup retained an increased proportion of higher risk individuals among Belgian patients, including multiple primary cancer patients (8/23; 34.8% vs. 6/63; 9.5%; *p* = 0.009) or first-degree relative(s) with breast and/or ovarian cancers (15/23; 65.2% vs. 23/63; 36.5%; *p* = 0.02; Figure 2C). We also evaluated the presence of “non-syndromic tumors” in the familial cancer history but their occurrence did not contribute to the increased frequency of PV.

In subgroups #1 and #2, the prevalence of PV in cancer predisposition genes for which an association with PDAC has not been established, was low (8/142 (5.6%)). The most frequent were PV in *CHEK2* (in three patients from PDAC families and in one colorectal cancer family). PV in *BRIP1, FANCM, NBN,* and *POLE* were identified only once each. One Czech PDAC patient harbored a PV in three genes: *CHEK2, ERCC4,* and *FANCG*.

Taken together, a family cancer history of PDAC or other syndromic tumors (in 30/35 patients with PV) represented the most important factor indicating a PV in a PDAC-predisposing gene.

Subgroups #3 and #4 included 149 sporadic (mainly Czech) PDAC patients. Only five (3.4%) of them harbored a PV in an established PDAC-predisposition gene, including 2 × *BRCA2*, 2 × *PALB2,* and 1 × *ATM*. Interestingly, the age at PDAC onset in these PV heterozygotes ranged between 59 and 71 years, while no PV in an established PDAC-predisposition gene was identified in the 52 patients diagnosed before the age of 59 years (Figure 2C).

The prevalence of PV in cancer predisposition genes for which an association with PDAC has not been established, was comparable for sporadic (11/149; 7.9%) and familial cases (subgroups #1 and #2: 8/142; 5.6%).

Our data indicate that PDAC patients without a family history of tumors associated with PDAC risk have a lower probability to harbor a clinically actionable PV.

### 3.4. Survival in Individuals with PV

The overall survival (OS) data were available for 223/226 Czech PDAC patients, including 31/33 patients with PV. The survival ranged between 0.3 and 281.1 months with mean OS 12.8 months for all PDAC patients. Baseline clinicopathological characteristics were similar for patients with and without PV (Table S6), except for the decreased proportion of tumors localized in the caput (having better survival) in patients with a PV in non-HR gene.

We first compared OS between patients with and without any PV (Figure 2D, left) and, secondly, with a PV in the genes coding for proteins involved in DDSB repair via HR pathway (*ATM, BRCA1/FANCS, BRCA2/FANCD1, BRIP1/FANCJ, ERCC4/FANCQ, FANCE, FANCG,* and *PALB2/FANCN*) and with PV in other non-HR genes (*CHEK2, HOXB13, NBN, POLD1,* and *TP53*), respectively (Figure 2D, right).

The risk of death was significantly reduced in patients with PV compared to patients without PV (HR = 0.66). Indeed, the mean OS (mOS) was 12.4 months in patients without PV versus 15.9 months in patients with PV. This improved survival was mainly associated with PV in HR genes because the presence of PV in non-HR genes actually led to worse OS (HR = 3.26). In only 4/22 (18.2%) of the patients with a pathogenic HR variant survival was shorter than the mOS (12.4 months), compared to 7/9 (77.8%) of the patients with a PV in a non-HR gene.

## 4. Discussion

Our study enabled a comparison of germline variations in unselected Czech and high-risk Belgian PDAC patients, for whom no studies have been published previously. The highest clinical utility is attributed to germline variants in genes known to be associated with PDAC predisposition (Table 2) [3]. The most frequently mutated genes in our study included *ATM*, *BRCA1*, and *BRCA2* (found in 25/35 (71.4%) patients with a PV in a PDAC-predisposition gene). *BRCA2* PV were the most frequent, accounting for 17% and 50% of all PV in PDAC-predisposing genes in Belgian and Czech patients, respectively. *BRCA2* and *BRCA1* code for proteins participating in the DNA DSB repair by HR and are the major genetic factors involved in hereditary breast/ovarian cancer syndromes. PV in both genes were associated independently with a high (OR > 5) and statistically significant PDAC risk in both populations. Interestingly, while in Belgian and Czech breast cancer patients the frequency of *BRCA1* germline PV is higher than *BRCA2*, *BRCA2* PV prevails in PDAC patients in both populations [19,20]. Thus, the *BRCA2*-associated risk could be higher for pancreatic than for breast cancer, as indicated by our analysis of pooled patients against gnomAD data (*BRCA1*: OR = 6.0 vs. *BRCA2*: OR = 11.7; Figure 1). Similar data were presented in the US study of 3030 PDAC patients by Hu and colleagues (OR 2.6 vs. 6.2) [21].

While *BRCA2* PV dominated in the Czech patients, *ATM* PV were the most frequent in Belgian patients (5/72; 6.9%). Biallelic *ATM* inactivation causes ataxia-telangiectasia while heterozygous variants moderately increase the risk of several tumors including breast or pancreatic cancers [22]. The frequency of *ATM* PV found in the Belgian subgroup is similar to observations in unselected Canadian PDAC patients (12/177; 6.8%) [23]. In the Czech PDAC patients, the frequency of germline *ATM* PV was lower (3/226; 1.32%) but still over three-times as high as in population-matched controls (Table 2). Interestingly, all eight *ATM* PV carriers in our study had a positive family cancer history (this may explain a significant association with the PDAC risk in the Belgian subgroup only, enriched in such patients). An aggregated analysis of all patients showed a high PDAC risk associated with *ATM* PV (OR = 5.6), comparable with that of *BRCA1*. Similar *ATM*-associated risks were calculated in Canadian (OR = 7.7) and US (OR = 5.7) PDAC patients [21,23].

PV in other PDAC-predisposition genes (*PALB2*, *CDKN2A,* and *TP53*) were substantially less frequent (5/35; 14.2% of PV carriers) and their significant associations with PDAC were not reached. However, PV in the mismatch repair genes (*MLH1*, *MSH6,* and *PMS2*) identified in five Belgian (but none in Czech) PDAC patients resulted in a significant association with PDAC collectively (and for *MLH1* and *PMS2* separately) in the Belgian subgroup. Generally, the prevalence of germline PV in mismatch repair genes in unselected PDAC patients is estimated at 0.3% to 1.3% [8,9,21,24].

Germline PV in cancer predisposition genes for which the association with PDAC has not been firmly established were less frequent. However, *CHEK2* PV accounted for 13.1% of all germline PV (identified in 8 of 298 PDAC patients (2.7%)), a proportion comparable to *ATM*. *CHEK2* was initially reported as a multi-organ cancer susceptibility gene associated with breast, prostate, colon, and pancreas cancer, with low-to-moderate penetrance [25]. Nevertheless, the majority of the clinical knowledge was adopted in pre-NGS era and only a few recurrently-analyzed *CHEK2* variants were included [26,27]. An increased frequency of *CHEK2* PV was reported in several PDAC studies but without statistical significance [10,21,28]. We found an increased population-specific PDAC risk for *CHEK2* PV in both Czech and Belgian cohorts and also when considered all patients together (Figure 1). In the three Belgian patients, c.1100delC was identified; the Czech *CHEK2* PV spectrum was more diverse, as recently reported in Czech breast cancer patients [15]. Interestingly, 3/5 PDAC patients (one Czech and two Belgians) with the c.1100delC also had one or more relatives diagnosed with PDAC. However, clinical classification of the *CHEK2* VUS (dominantly missense variants and short in-frame indels) as well as larger case-control studies in other populations are warranted to further analyze the *CHEK2* association with PDAC risk.

The *ERCC4* gene was the second most frequently mutated gene among genes with uncertain PDAC risk. Germline *ERCC4* inactivations causes xeroderma pigmentosum complementation group F, Cockayne syndrome, or Fanconi anemia complementation group Q [29]. Japanese patients homozygous for *ERCC4* p.Arg799Trp or compound heterozygous with another variant were diagnosed with autosomal recessive cerebellar ataxias [29]. Heterozygous carriers were recently described in PDAC patients from the USA and the authors recommended to include *ERCC4* into the germline panel testing [11]. We found p.Arg799Trp to be present in 4/226 (1.34%) Czech PDAC patients but also in 5/777 (0.64%) population-matched controls, making the risk in Czech patients insignificant.

The c.657_661del variant in *NBN* represented another example of a variant prevailing in Czech patients. An association of this variant with PDAC was previously documented in Polish patients and in an independent Czech PDAC cohort [30,31]. In the current study, the c.657_661del variant was more frequent in Czech PDAC patients than in the population-matched controls; however, the difference was not significant. The *ERCC4* and *NBN* variants exemplify the need for careful analysis of risk, associated with population-specific or founder variants, using appropriate geographically-matched controls [32]. However, the insignificant differences in the overall frequency of PV in non-PDAC established cancer predisposition genes among subgroups of patients considering personal/family cancer history or population origin indicate that the roles of many these genes in PDAC predisposition is limited.

Considering the clinical characteristics of patients with PV, our study demonstrated that a family cancer history is an important risk factor for the identification of a germline PV in PDAC-predisposition genes in both populations. The PDAC patients with first or second degree relatives developing pancreatic/breast/ovarian/colorectal cancer or melanoma have a much higher chance to have a PV in a PDAC-predisposition gene compared to the patients without a family cancer history (OR 7.7; *p* = 2.6 × 10^−6^; Table 3).

The difference in PV frequencies in PDAC-predisposition genes between Czech (8.0%) and Belgian (23.6%) PDAC patients were attributable to an ascertainment bias towards high-risk PDAC patients in the Belgian subgroup. The overall PV frequency in unselected Czech PDAC patients (8.0%) corresponded to studies from unselected PDAC patients in the USA (7.7%) [24], or Canada (10.7%) [23]. The frequency of PV in Belgian high-risk PDAC patients insignificantly exceeded the frequencies reported in high-risk PDAC patients from the USA (17.7%) [33] or familial PDAC patients from Germany (16.7%) [34].

To leverage the differences in enrollment of high-risk individuals in Belgian and Czech cohorts, we assigned all patients into subgroups reflecting their family cancer history or PDAC age of diagnosis. The subsequent analysis revealed similar frequencies of PV in PDAC-predisposition genes in Belgian and Czech familial PDAC patients (subgroup #1; 26.5% and 27.3%, respectively). These frequencies were higher than in published studies from Germany (16.7%) [34], the USA (11.9%) [35], and Japan (14.8%) [36]. However it is important to note that there is no uniform definition of familial PDAC and many different working definitions are being used [6,37].

We observed that double primary PDAC/another tumor from breast/ovarian/colorectal/melanoma hereditary cancer spectrum strongly predicts the presence of a germline PV in a PDAC-gene, as described previously [33].

We found no effect of PV presence on the age of PDAC onset, in agreement with previous reports from Canada and the USA [38,39] but in contrast to two other US studies [7,40]. These contradictory results indicate that the association of germline PV with age of onset remains inconclusive. Most experts in current CAPS guidelines do not recommend screening of high risk individuals before the age of 50 with the exception of patients with Peutz-Jeghers syndrome or hereditary pancreatitis [4].

Clinical data from Czech PDAC patients confirmed a positive impact of germline PV, especially in HR genes, on the survival of PDAC patients (21.4 months vs. 12.4 months, Figure 2D). Similar data were reported from Goldstein et al. (17.9 vs. 9.6 months) [11], Fountzilas et al. (22.6 vs. 13.9 months) [10] or Yurgelun et al. (34.4 vs. 19.1 months) [9]. An association of germline PV in non-HR genes with inferior survival (4.7 vs. 12.4; HR = 3.26; *p* = 0.017) needs to be further evaluated because our group consisted of only nine individuals enriched in tumors with prognostically-inferior localization in the pancreas.

The identification of germline PV associated with an increased PDAC risk has important implications for both patients and their relatives. The predictive role of germline PV include application of programmed death receptor–1 (PD-1) pathway inhibitors (in patients with mismatch repair deficiencies) or PARPi (in patients with germline PV in HR genes) [13,41].

There are some limitations to this study. The ascertainment criteria differed between Czech and Belgian patients. The family history of the PDAC patients relied on self-reported family history of PDAC and other cancers, potentially biasing an inaccuracy of medical history. Although multigene panel testing is an effective and cost-effective strategy to identify PV in various genes, the clinical interpretation of cancer risk for multiple moderate penetrance genes remains challenging. To allow formulating consensus guidelines for medical management of the PV carriers, additional large studies with detailed information on personal and family history are definitively warranted.

## 5. Conclusions

Our study demonstrated that germline PV in *BRCA2*, *ATM,* and *BRCA1* are the most frequent in both Belgian and Czech PDAC patients and we confirmed that these are associated with a significantly increased PDAC risk. PV are more frequent among PDAC patients with multiple primary tumors and/or with a positive family history of PDAC or breast/ovarian/colon cancer or melanoma. The presence of PV in *BRCA2*, *ATM*, *BRCA1,* and other HR genes was associated with improved OS in PDAC patients and entails clinically useful prognostic information. Therefore, clinical germline genetic testing of genes increasing the PDAC risk (including *BRCA2*, *ATM*, *BRCA1*, *PALB2*, *MLH1*, *MSH2*, *MSH6*, *PMS2*, *CDKN2A*, *TP53*, *STK11,* and possibly also *CHEK2*) should be offered to all PDAC patients or at least to those with a positive family cancer history or personal history of multiple primary tumors. Beyond the prognostic information, the identification of a germline PV in PDAC patients bears a predictive value enabling tailored anticancer treatment using platinum chemotherapy or PARPi. Moreover, the cascade testing in relatives and intensified cancer surveillance in carriers of a particular PV in a cancer-predisposition gene represent an important approach reducing cancer burden in these high-risk individuals. Our study also highlights the importance of a population-matched control population for establishing correct risk associations.

## Figures and Tables

**Table 1:** Clinical characteristics of analyzed PDAC patients.

Characteristics	All Patients (*n* = 298)	Belgian Patients (*n* = 72)	Czech Patients (*n* = 226)	*p*-Value *
Gender				
Female (%)	150 (50.3)	41 (56.9)	109 (48.2)	0.22 a
Male (%)	148 (49.7)	31 (43.1)	117 (51.8)	
Age at PDAC diagnosis; Mean age (SE)	61.9 (0.6)	58.0 (1.4)	63.11 (0.6)	0.002 b
<50 (%)	40 (13.4)	19 (26.4)	21 (9.3)	
50–59 (%)	77 (25.8)	17 (23.6)	60 (26.6)	
60–69 (%)	120 (40.3)	26 (36.1)	94 (41.6)	
≥70 (%)	61 (20.5)	10 (13.9)	51 (22.6)	
Multiple primary tumors in personal history				
Absent (%)	260 (87.2)	51 (70.8)	209 (92.5)	<0.0001 a
Present (%)	38 (12.8)	21 (29.2)	17 (7.5)	
Multiple primary tumors in personal history				
Breast (%)	19 (6.4)	11 (15.3)	8 (3.5)	0.001 a
Ovarian/endometrial (%)	6 (1.7)	2 (2.8)	4 (1.8)	0.63 a
Colon (%)	7 (2.3)	7 (9.7)	0	<0.0001 a
Melanoma (%)	3 (1.0)	3 (4.2)	0	0.01 a
Other (%)	12 (4.0)	4 (5.6)	8 (3.5)	0.73 a
Family cancer history ^‡^ (first/second-degree relatives)				
Negative (%)	149 (51.2)	9 (13.6)	140 (62.2)	<0.0001 a
Positive (%)	142 (48.8)	57 (86.4)	85 (37.8)	
Unknown	7	6	1	
Syndromic ^‡^ tumors in family cancer history				
Pancreatic (%)	56 (19.2)	34 (51.5)	22 (9.8)	<0.0001 a
Breast (%)	59 (20.3)	27 (40.9)	32 (14.2)	<0.0001 a
Ovarian/endometrial (%)	13 (4.5)	4 (6.1)	9 (4.0)	0.50 a
Colon (%)	52 (17.9)	16 (24.2)	36 (15.9)	0.14 a
Melanoma (%)	7 (2.4)	3 (4.5)	4 (1.8)	0.19 a

a Fisher exact test, b Welch *t*-test, NA—not available, * Belgian vs. Czech subgroups. **^‡^** Fulfilling criteria for hereditary breast and ovarian cancer syndrome (HBOC), familial adenomatous polyposis (FAP) or familial atypical multiple mole melanoma (FAMMM). The considered tumors included PDAC, colorectal, breast, ovarian cancer, and melanoma.

**Table 2:** Prevalence of pathogenic/likely pathogenic variants (PV) in Belgian/Czech patients and controls.

	PDAC Patients			Population-Matched Controls	
Germline PV	All; *n* = 298	Belgian; *n* = 72	Czech; *n* = 226	Belgian; *n* = 2485	Czech; *n* = 777
Known PDAC-Predisposition Genes					
ATM *	8 (2.68%)	5 (6.94%)	3 (1.32%)	7 (0.28%)	3 (0.39%)
*BRCA1*	5 (1.67%)	2 (2.78%)	3 (1.32%)	5 (0.20%)	1 (0.13%)
BRCA2 *	12 (4.01%)	3 (4.17%)	9 (3.98%)	15 (0.60%)	5 (0.64%)
*CDKN2A*	1 (0.33%)	1 (1.39%)	0	0	0
MLH1 *	3 (1.00%)	3 (4.17%)	0	1 (0.04%)	0
*MSH2*	0	0	0	2 (0.08%)	3 (0.39%)
MSH6 *	1 (0.33%)	1 (1.39%)	0	2 (0.08%)	0
*PALB2*	2 (0.67%)	0	2 (0.88%)	4 (0.16%)	2 (0.26%)
*PMS2*	2 (0.67%)	2 (2.78%)	0	4 (0.16%)	0
*STK11*	0	0	0	1 (0.04%)	0
*TP53*	2 (0.67%)	1 (1.39%)	1 (0.34%)	1 (0.04%)	0
PDAC gene PV	36	18	18	42	14
Number of individuals with PDAC PV *	35 * (11.74%)	17 * (23.61%)	18 (7.96%)	42 (1.69%)	14 (1.80%)
Other cancer predisposition genes for which the association with PDAC is not firmly established					
*BARD1*	0	0	0	1 (0.04%)	0
*BLM*	0	0	0	3 (0.12%)	3 (0.39%)
*BRIP1*	2 (0.67%)	0	2 (0.88%)	1 (0.04%)	0
*CDK4*	0	0	0	1 (0.04%)	0
*CHEK1*	0	0	0	1 (0.04%)	0
CHEK2 *	8 (2.68%)	3 (4.17%)	5 (2.21%)	11 (0.40%)	1 (0.13%)
ERCC4 *	4 (1.34%)	0	4 (1.76%)	6 (0.24%)	5 (0.64%)
*FANCA*	0	0	0	6 (0.24%)	3 (0.39%)
*FANCC*	0	0	0	1 (0.04%)	0
*FANCD2*	0	0	0	3 (0.12%)	1 (0.13%)
FANCE *	2 (0.67%)	1 (1.39%)	1 (0.44%)	2 (0.08%)	0
FANCG *	1 (0.33%)	0	1 (0.44%)	3 (0.12%)	0
*FANCI*	0	0	0	3 (0.12%)	1 (0.13%)
*FANCL*	0	0	0	1 (0.04%)	0
*FANCM*	1 (0.33%)	1 (1.39%)	0	10 (0.40%)	4 (0.51%)
*HOXB13*	1 (0.33%)	0	1 (0.44%)	7 (0.28%)	0
*MRE11*	0	0	0	5 (0.20%)	2 (0.26%)
*NBN*	3 (1.00%)	0	3 (1.32%)	7 (0.28%)	3 (0.39%)
*POLD1*	1 (0.33%)	0	1 (0.44%)	1 (0.04%)	0
*POLE*	1 (0.33%)	1 (1.39%)	0	1 (0.04%)	0
*PTEN*	0	0	0	1 (0.04%)	0
*RAD50*	0	0	0	3 (0.12%)	1 (0.13%)
*RAD51C*	0	0	0	1 (0.04%)	0
*RAD51D*	0	0	0	2 (0.08%)	0
*RAD54L*	0	0	0	7 (0.28%)	2 (0.26%)
*RECQL*	0	0	0	3 (0.12%)	3 (0.39%)
SLX4 *	1 (0.33%)	1 (1.39%)	0	6 (0.24%)	1 (0.13%)
*XRCC2*	0	0	0	1 (0.04%)	0
Other gene PV	25	7	16	98	30
Number of individuals with PV in other genes *	23* (7.72%)	7 (9.72%)	16 (7.08%)	98 (3.94%)	30 (3.86%)
All PV	61	25	36	148	45
All PV carriers *	54 * (18.12%)	21 * (29.17%)	33 * (14.60%)	140 (5.63%)	45 (5.79%)

* Multiple germline PV were found in 4 Belgian and 2 Czech PDAC patients with co-occurring PV in PDAC predisposition genes (MLH1-MSH6), in PDAC predisposition and other genes (ATM-FANCE; ATM-SLX4; BRCA2-CHEK2; BRCA2-ERCC4), or in the other genes (CHEK2-ERCC4-FANCG). The frequency of PV in all 4 Lynch syndrome genes was significantly increased in Belgian PDAC patients over Belgian controls (5/72 individuals (6 PV as 1 patient had a PV in both MLH1 and MSH6); 6.94% vs. 9/2485; 0.36%; OR = 20.5; 95%CI 6.7–62.6; *p* = 2.9 × 10^−11^).

**Table 3:** Prevalence of PV in four subgroups ascertaining 291 patients with known family cancer history.

PDAC Patients Group	All (*n* = 291); *n*	Patients with PV		Patients without PV *n* (%)	*p*-Value ^2^
		in PDAC Gene ^1^; *n* (%)	in Other Gene Only; *n* (%)		
Familial cancer patients	142	30 (21.1)	8 (5.6%)	104 (73.2)	
Belgian	57	17 (29.8)	4 (7.0)	36 (63.2)	0.07
Czech	85	13 (15.3)	4 (4.7)	68 (80.0)	
Subgroup#1: ≥1 PDAC in first/second degree relative	56	15 (26.8)	4 (7.1)	37 (66.1)	
Belgian	34	9 (26.5)	3 (8.8)	22 (64.7)	0.83
Czech	22	6 (27.3)	1 (4.5)	15 (68.2)	
Subgroup#2: ≥1 Tumor associated with increased PDAC risk in First/second degree relative	86	15 (17.4)	4 (4.7)	67 (77.9)	
Belgian	23	8 (34.8)	1 (4.3)	14 (60.9)	0.04
Czech	63	7 (11.1)	3 (4.8)	53 (84.1)	
Sporadic PDAC patients ^3^	149	5 (3.4)	11 (7.4)	133 (89.3)	
Belgian	9	0	0	9 (100)	n.d.
Czech	140	5 (3.6)	11 (7.9)	124 (88.5)	
Subgroup#3: Sporadic PDAC, early onset (≤60 years)	53	1 (1.9)	4 (7.5)	48 (90.6)	
Belgian	9	0	0	9 (100.0)	n.d.
Czech	44	1 (2.3)	4 (9.1)	39 (88.6)	
Subgroup#4: Sporadic PDAC, later onset (>60years)	96	4 (4.2)	7 (7.3)	85 (89.6)	
Belgian	0	0	0	0	n.d.
Czech	96	4 (4.2)	7 (7.3)	85 (89.6)	
Sum	291	35	19	237	

^1^ The patients with a PV in both a PDAC-predisposing and other gene were considered in the group of PV in PDAC genes. ^2^ Proportion of Belgian vs. Czech PV in a group. ^3^ Including patients with other tumors in family cancer history (not associated with PDAC-predisposition syndromes). The family cancer history was not detailed enough for 6 Belgian patients and 1 Czech PDAC patient. n.d.—not determined.

## Data Availability

Data sets are available upon request to the corresponding authors (Kathleen Claes and Marketa Janatova) and can be shared after consulting our local institutional review boards.

## Supplementary Materials

The following are available online at https://www.mdpi.com/article/10.3390/cancers13174430/s1, Table S1: Overlapping genes between both panels; Table S2: List of 226 genes Czech CZECANCA panel; Table S3: List of 66 targeted genes in Belgian SeqCAP panel; Table S4: List of all the (likely) pathogenic variants in PDAC patients; Table S5: Comparison of pathogenic variant frequencies between PDAC cases (BEL + CZE) and control data; Table S6: Baseline characteristics comparing mutation carriers (in HR and non-HR genes) collectively and separately with mutation non-carriers.
